# Advanced Cases of Tuberculosis

**Published:** 1919-10-04

**Authors:** 


### ADVANCED CASES OF TUBERCULOSIS.

### Compulsory Removal Proposed.

Representations are to be made to the Ministry of Health to extend the scope of the Tuberculosis Regulations of 1911 in order to provide that a medical officer of health shall, after consultation with a medical practitioner, have power to order the compulsory removal of cases of consumption where it can be shown that the patients are a danger to the public health. This proposal emanates from the Southwark Borough Council, the first public authority in the country to provide out of the rates accommodation in the country for tuberculosis patients.

Dr. G. Millson, the Medical Officer of Health, in conjunction with the Town Clerk (Mr. Percy H. Gray) and the Tuberculosis Officer (Dr. Wilson), were asked by the Public Health Committee to consider this matter, and in their report they point out that the compulsory power of removal by local authorities in respect of certain cases of infectious disease already exist under the Public Health (London) Act, 1891, but this does not extend to tuberculosis. Article 7 of the Tuberculosis (in Hospital) Regulations, 1911, provides that nothing in the regulations shall authorise or require a medical officer of health or a local authority to put in force any enactment which renders a consumptive or the person in charge of the patient or any other persons liable to a penalty, or subjects the patient to any restriction, prohibition or disability affecting himself or his employment, occupation or means of livelihood on the ground of his suffering from tuberculosis.

Compulsory powers of removal have, however, been obtained by some of the provincial councils under private Acts of Parliament, *e.g.*, the St. Helens Corporation Act of 1908.

The Public Health (Prevention and Treatment of Disease) Act, 1913, also contains a special clause dealing with treatment of tuberculosis. Section 3 empowers any sanitary authority to make any such arrangement as may be sanctioned by the Local Government Board (now the Ministry of Health) for the treatment of tuberculosis: this power to be in addition to and not in derogation of any other power.

If this section be acted upon it may happen that one borough will go in for compulsory removal of advanced consumptives, whilst another may not act so drastically. They are of opinion, having regard to the fact that a certain percentage of consumptive cases are a danger and cause of infection to others, and refuse to enter an institution or take steps to prevent the spread of the disease that compulsory removal should be general throughout the country in proper cases. They are therefore of opinion that the Tuberculosis Regulations of 1911 should be so extended that a medical officer of health shall have power to order with due legal precautions the compulsory removal of a case where it is clearly proved that such a case is tending to spread the disease, and the affected person refuses to take advantage of voluntary measures offered.

The same gentlemen also considered the question of the provision by the State of accommodation other than that provided by institutions maintained by voluntary charitable bodies, in which persons in an advanced stage of consumption can end their days. In this connection they point out that as far back as 1913, and again in 1918, conferences of London sanitary authorities expressed the strong opinion that greater attention should be paid to the treatment of advanced and incurable cases of tuberculosis.

The only State provision provided at present is accommodation in a Poor-Law institution, and a number of beds for discharged soldiers provided by arrangement with the Insurance Commissioners, but very few consumptives will voluntarily enter a workhouse infirmary. The accommodation provided by voluntary institutions for such cases is quite inadequate for the need. It is generally admitted that the segregation and housing of advanced cases should be undertaken by the local authorities, and accommodation provided free from the pauper taint so much dreaded by the unfortunate patients.

The Southwark Borough Council was asked to approach the London County Council to take over and maintain as a "rest home" for persons in an advanced stage of consumption the Red Cross Hospital at Kennington Park Road, which was opened under the auspices of the Borough Council, and maintained by public subscriptions, but unfortunately this scheme had to be withdrawn, as the premises had been secured for another purpose, whilst the lessees, it was stated, objected to the premises being used for this purpose. Undaunted by this rebuff, the Public Health Committee are endeavouring to find other premises which could be converted into a "rest home" for these advanced cases of tuberculosis.

